# Recent Advances in the Application of CRISPR/Cas9 Gene Editing System in Poultry Species

**DOI:** 10.3389/fgene.2021.627714

**Published:** 2021-02-19

**Authors:** Collins N. Khwatenge, Samuel N. Nahashon

**Affiliations:** ^1^Department of Biological Sciences, Tennessee State University, Nashville, IN, United States; ^2^Department of Agriculture and Environmental Sciences, Tennessee State University, Nashville, TN, United States

**Keywords:** CRISPR/Cas9 system, genome editing, transgenic, gene editing, poultry species, primordial germ cells

## Abstract

CRISPR/Cas9 system genome editing is revolutionizing genetics research in a wide spectrum of animal models in the genetic era. Among these animals, is the poultry species. CRISPR technology is the newest and most advanced gene-editing tool that allows researchers to modify and alter gene functions for transcriptional regulation, gene targeting, epigenetic modification, gene therapy, and drug delivery in the animal genome. The applicability of the CRISPR/Cas9 system in gene editing and modification of genomes in the avian species is still emerging. Up to date, substantial progress in using CRISPR/Cas9 technology has been made in only two poultry species (chicken and quail), with chicken taking the lead. There have been major recent advances in the modification of the avian genome through their germ cell lineages. In the poultry industry, breeders and producers can utilize CRISPR-mediated approaches to enhance the many required genetic variations towards the poultry population that are absent in a given poultry flock. Thus, CRISPR allows the benefit of accessing genetic characteristics that cannot otherwise be used for poultry production. Therefore CRISPR/Cas9 becomes a very powerful and robust tool for editing genes that allow for the introduction or regulation of genetic information in poultry genomes. However, the CRISPR/Cas9 technology has several limitations that need to be addressed to enhance its use in the poultry industry. This review evaluates and provides a summary of recent advances in applying CRISPR/Cas9 gene editing technology in poultry research and explores its potential use in advancing poultry breeding and production with a major focus on chicken and quail. This could aid future advancements in the use of CRISPR technology to improve poultry production.

## Introduction: Gene Editing Tools

The poultry industry is undergoing a gene editing revolution that will change the poultry genome in the near future through targeted gene editing of the poultry species (Hwang and Han, 2018). The application of genome editing technology in the poultry industry, as well as livestock production in general, has improved over the last decade due to the availability of precision genome engineering tools (Petersen, 2017; Cooper et al., 2018). There are three commonly used genome-editing techniques for the production of animals, including poultry. The first is the zinc finger nuclease (ZFNs), which is used for binding specific DNA domains that complement the target DNA sequences. Secondly, transcription activator-Like effector nucleases (TALENs) are another gene and genome editing technology that employs the nuclease domain to produce double strands breaks (DSBs). Finally, yet importantly, the clustered regularly interspaced short palindromic repeats (CRISPR)-associated protein 9 (CRISPR/Cas9), is the most common and advanced technique for genome editing. The similarity between these three techniques is that they all require the two domains for accurate and defectless gene and genome editing. ZFN and TALEN differ from CRISPR/Cas9 since both use proteins that are fused together as a DNA binding domain while the CRISPR/Cas system requires the use of a specific RNA sequence molecule for DNA binding instead of the fused proteins (Kim and Kim, 2014; Razzaq and Masood, 2018). ZFNs and TALENs also require more time to produce an effective system, making the two more-time consuming. ZFNs and TALENs have been found to have more off target effects as opposed to CRISPR/Cas9 system (Hwang and Han, 2018; Bahrami et al., 2020). This is because of the availability of computational tools while using the CRISPR/Cas9 system that help in designing sgRNAs. Therefore, predictability of guide specificity is achieved, and this minimizes off-target effects. There is also a chance that the design of successful sgRNAs with the available CRISPR/Cas9 computational tools has a strong on-target activity hence reducing off-target effects (Wilson et al., 2018). The CRISPR/Cas9 technology uses a specific RNA sequence called guide RNA which binds to another target sequence of DNA (target DNA) followed by the cleavage of Cas9 where binding has occurred. This makes the CRISPR/Cas9 system stand out as the most suitable gene editing tool as it improves the frequency of precise genome modifications in creating genetically edited animals (Chu et al., 2015). The CRISPR-based system is continuously undergoing improvement. The most recent development of the CRISPR system employs coexpression of CRISPR-associated nucleases 9 and 12a hence having the ability to edit multiple target sites in the genome at the same time to help study how different genes cooperate in functions (Pennisi, 2013). Therefore, this system is very important in interrogating gene functions (Cong et al., 2013; Yang et al., 2013; Najm et al., 2018; Gonatopoulos-Pournatzis et al., 2020).

CRISPR is a family of DNA sequences found in the genomes of prokaryotic organisms such as bacteria and archaea. These sequences are derived from DNA fragments of bacteriophages that had previously infected the prokaryote. The CRISPR tool together with Cas endonuclease is a powerful programmable nuclease system (Barrangou et al., 2007). Studies conducted by Jinek et al. (2012) unveiled a double RNA, known as a guide RNA (gRNA) which consisted of a 20-bp CRISPR RNA (crRNA) and universal trans-activating crRNA (tracrRNA). This RNA coupled with *Streptococcus pyogenes* type II Cas9 protein can induce cleavage of specific target DNA sequences in virtually any organism. The Cas9 nuclease activity is initiated by protospacer adjacent motif (PAM) sequence NGG, which is usually located next to the target site (Anders et al., 2014). It is possible to engineer DNA Cas9-mediated DSBs at a specific genomic locus. Non-homologous end-joining (NHEJ) can induce DSB repair that disrupts the target gene, generating insertions and deletions. Another way of repairing Cas9-mediated DSBs is by homologous directed repair (HDR), which allows specific gene editing by integrating genetic modifications into the target template (Thomas and Capecchi, 1987; Salsman and Dellaire, 2017).

## The Status of CRISPR/Cas9 Technology in the Poultry Industry

The CRISPR/Cas9 system is among the gene editing technologies that are creating a rapid change in poultry genomics for both poultry breeding and food production purposes (Doran et al., 2017). To date, substantial progress in using CRISPR/Cas9 technology has been made in only two poultry species (chicken and quail), with chicken taking the lead. The CRISPR technology is not aimed at replacing the traditional breeding system, but it provides a complementary option by giving the breeder more genetic variation to select from since the use of traditional breeding for genetic gain has limitations of introducing genetic variation within a given population of the poultry flock. The introduction of genetic variations using the CRISPR/Cas9 system can be used to improve the performance of livestock animals such as poultry.

The CRISPR/Cas9 system has several benefits that could be used to improve poultry growth and production performance. These benefits include increased bird performance by improving the digestibility and overall growth, increased egg production, increased bird’s immunity and disease resistance, producing birds that are leaner with little or no fat deposition in poultry meat for better nutritional profiles. A good example is the recent attempt to create chickens that have decreased accretion of abdominal fat and increased lean percentage of carcass meat by altering the percentage of fatty acid composition (Park et al., 2019). The CRISPR/Cas9 has also been employed in animal welfare improvements through in-ovo sexing (Lee et al., 2019b). There is an increased need to produce birds that meet the benefits of both commercial producers and consumers in the poultry industry. Several strategies have been proposed for the generation of transgenic birds to meet several demands in the poultry industry. This review discusses various applications of the CRISPR/Cas9 technology for genome editing in poultry, with a focus on recent and current advances in CRISPR/Cas9-mediated gene editing technology to produce genetically modified birds for various purposes. This review also provides a summary and discussion of the challenges, possible approaches, and future perspectives on applying CRISPR/Cas9 technology for gene and genome engineering in poultry species.

## Generation of Genetically Modified CRISPR/Cas9-Mediated Birds

CRISPR/Cas9 has gained traction as an efficient method for precise gene editing and modification of genomes in various organisms including the avian species (Bai et al., 2016; Oishi et al., 2016; Wang et al., 2017b). Various methods have been proposed to produce genetically modified animals. In mammals, germ-line modification was used in the generation of the first transgenic animals such as mice, rabbits, sheep, and pigs, by microinjection of the target DNA into the pro-nucleus of a fertilized embryo (Gordon et al., 1980; Hammer et al., 1985). Another method that has been used to modify the germ line in animals uses embryonic stem cells (ESCs). ESCs are genetically modified, then cells are injected into the recipient blastocyst to produce germ-line chimeras. Unlike mammals, the microinjection of avian ESCs into the zygote in avian species is very difficult because the avian zygote is surrounded by a large amount of yolk and a small germinal disc. Therefore, the first transgenic chicken was produced *via* retroviral injection into the sub-germinal cavity of Eyal-Giladi and Kochav (EGK; Eyal-Giladi and Kochav, 1976) stage X embryos (Salter et al., 1986). Salter et al. (1987) created the first retrovirus-mediated transgenic chickens by insertion of retroviral genes into the chicken germ line. Their transmission frequencies varied from 1 to 11%. McGrew et al. (2004) produced germline transgenic chickens using lentiviral vectors with transmission efficiencies between 4 and 45%. Lillico et al. (2007) generated the first oviduct-specific expression of transgenes in hens but there was very low efficiency in the rate at which transgenic birds were generated. Various strategies such as the viral infection of stage X embryos (Thoraval et al., 1995; Sherman et al., 1998), microinjection of transgenes into fertilized eggs (Love et al., 1994; Sherman et al., 1998), and embryonic stem cells (Zhu et al., 2005) have been used to produce transgenic birds. In van de Lavoir et al. (2006) generated the first inter-individual transfer of chicken primordial germ cells (PGCs). As compared to the use of ESCs in mammals, PGCs have been used widely in the generation of transgenic birds to overcome the limitation of low efficiency germ-line transmission. Transgenes can be introduced into the cultured genomes of PGCs using transfection reagents to produce transgenic birds (Han and Park, 2018). Transgenic birds have been generated by injection of transgenes into the embryonic blood vessel to transfect the circulating PGCs to produce germline chimera, although these birds had a lower transgenic efficiency (Zhang et al., 2012; Tyack et al., 2013; Lambeth et al., 2016). Just before the onset of the CRISPR technology, Schusser et al. (2013) created the first knock-out in chickens using efficient homologous recombination in primordial germ cells.

**Figure 1:**
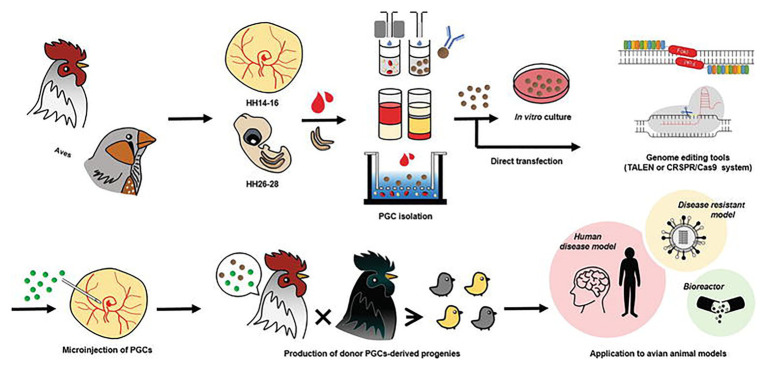
Genome editing in poultry species. Primordial germ cells (PGCs) in poultry can be obtained from embryonic blood and embryonic gonads. Delivery of genome editing tools such as the CRISPR/Cas9 system is followed by the establishment of genome-edited poultry by microinjection of directly isolated or *in vitro* cultured PGCs into the blood vessels of recipient embryos. Avian genome editing systems can be applied to produce various avian models and poultry. This figure is reproduced from an earlier publication (Han and Park, 2018, p. 19) after obtaining the permission from Journal of Animal Science and Biotechnology and the corresponding author (Jae Yong Han, Seoul National University, Seoul, Korea).

With the advent of the CRISPR/Cas9 system, an *in vitro* culture system for PGCs can be combined with this efficient genome-editing system to produce programmable genome-edited poultry. First, the PGCs in poultry can be obtained from embryonic blood or gonads. The delivery of the CRISPR/Cas9 system is followed by the establishment of genome-edited poultry by the microinjection of directly isolated or *in vitro* cultured PGCs into the blood vessels of recipient embryos to produce a chimera that hatches and grows into mature avian poultry. Oishi et al. (2016) used the CRISPR/Cas9 system to efficiently generate ovomucoid gene-targeted chickens by transferring transiently drug-selected PGCs into recipient embryos using gamma-ray irradiation to deplete endogenous PGCs. In one of their most recent works, CRISPR/Cas9-mediated knock-in of human interferon beta (hIFN-β) was created into the chicken exon 2 of the ovalbumin gene (Oishi et al., 2018). Since the generation of the first CRISPR/Cas9-mediated chicken in 2015 by Veron and his group (Véron et al., 2015) through electroporation of chicken embryos, many more studies involving transgenic poultry-related species have been published as discussed in the next section. The current trend in using the CRISPR/Cas9 system in poultry species is incorporating this genome editing tool with genomic analysis software such as CRISPR to increase target specificity, efficiency, and lower off-target effects. Figure 1 shows a workflow using the CRISPR/Cas9 system of programmable genome editing in avian species.

## CRISPR/Cas9-Mediated Genome Editing in Selected Poultry Species

Many researchers are studying the potential use of CRISPR/Cas9 for genome editing in the avian species. There is substantial progress in using CRISPR/Cas9 technology in chicken and quail, with chicken taking the lead as far as the poultry industry is concerned. Véron et al. (2015) published the first CRISPR/Cas9-mediated chickens 5 years ago. This study coupled the use of electroporated chicken embryos with Cas9 and guide RNAs encoded plasmids against the transcription factor paired box 7 (PAX7). In another recent study, the CRISPR/Cas9 system was used to produce chicken using ovalbumin and ovomucoid (OVM) genes. In this study, puromycin-selected CRISPR-induced mutant-ovomucoid PGCs were transiently transplanted into recipient chicken embryos with gamma-ray irradiation (Oishi et al., 2016). Their results indicated that the CRISPR/Cas9 system was used to induce OVM mutation getting a high efficiency (93%) in most donor PGCs with an average mutant semen efficiency of 93%. Another study in chicken by Dimitrov et al. (2016) shows a successful germline gene editing by efficient CRISPR-mediated homologous recombination in primordial germ cells. In this study, an additional *loxP* site was inserted into the variable region segment of a *loxP* by homology directed repair (HDR). This segment had been previously inserted into the chicken immunoglobulin heavy chain (IgH) locus gene. Their results showed variable germline transmission rates (0–90% efficiency) for the different PGC lines used.

As studies, PGC lines show different germline competencies for genetic modification and gene editing using CRISPR/Cas9 technology (Naito et al., 2015). More recently, Cooper et al. (2017) also reported a very successful method of avian genome editing known as “sperm transfection-assisted gene editing.” This method involves the delivery of CRISPR gRNA and Cas9 mRNA mixture directly into a mature chicken sperm cell. This method was able to achieve a targeting efficiency of 26.6% and about 3% mutation in the green fluorescent protein (GFP) and, double sex and mab-3 related transcription factor 1 (DMRT1) genes, respectively. Morin et al. (2017) have recently described a technique that combines the CRISPR/Cas9 system with *in vivo* electroporation hence inhibiting the gene functions of target genes in the somatic cells of developing chicken embryos.

Abu-Bonsrah et al. (2016) worked on projects that targeted genes in the DF-1 and DT-40 cell lines. The genes targeted are highly important in embryonic progression for targeted genetic manipulation of the chicken genome using the CRISPR/Cas9 system. These genes included EZH2, CDKN1B, DROSHA, MBD3, KIAA1279, HIRA, TYRP1, among others. Many methods for CRISPR/Cas9-mediated gene modifications in avian species are based on genome modification of PGCs *in vitro* followed by in-ovo injection of modified PGCs into the embryonic blood vessels. There is however a possibility of using adenoviral vectors for delivery of CRISPR/Cas9 into the bird blastoderm in eggs resulting in chimeras that generate offspring having targeted mutations (Lee et al., 2019c). This technique of generating genome-edited poultry could fast-track many avian research studies with potential applications in poultry production. The use of poultry-specific CRISPR/Cas9 designed vectors containing inserted avian-specific promoters for the expression of guide RNA and Cas9 protein can efficiently introduce targeted gene modifications in poultry species (Ahn et al., 2017). This type of CRISPR vector can be applied in many poultry species to generate efficient knockout avian cell lines and knockout birds for various purposes.

Quail is an important avian species due to its value in the poultry food industry and its use as a research model for various research areas, especially avian transgenesis and genome editing. Currently, the use of CRISPR/Cas9 genome editing technology is more widely used in chicken than quail since chicken has been the most valuable avian model in developmental biology and immunology. Quail is however gaining tract as an alternative model to chicken in genome-editing studies due to their short generation time, high level of producing eggs, and small size (Poynter et al., 2009; Lee et al., 2019c). Ahn et al. (2017) designed a poultry-specific CRISPR/Cas9 system that introduces targeted deletion mutation in chromosomes of the quail muscle cell lines using a customized quail CRISPR vector. In this study, quail 7SK promoter and CBh promoter were cloned into a CRISPR vector for the expression of gRNA and Cas9 protein. The gRNA was designed to target the quail melanophilin (MLPH) locus. Lee et al. (2019c) reported CRISPR/Cas9-mediated gene knockouts in quail targeting the MLPH gene. In this study, CRISPR/Cas9 adenoviral vector was directly injected into the quail blastoderm. The offspring obtained from the quail chimeras were found to have mutations in the MLPH gene. Lee et al. (2020) targeted the myostatin (MSTN) gene to generate mutations in quail *in vivo* using an adenoviral CRISPR/Cas9 system-mediated method. This study showed that the mutation in MSTN resulted in the deletion of cysteine 42 in the MSTN propeptide region and the homozygous mutant quail showed significantly increased body weight and muscle mass decreased fat percentage weight and increased heart weight as compared to heterozygous mutant and wild-type quail.

## Applications of CRISPR/Cas9 System in Poultry-Related Species

**Figure 2:**
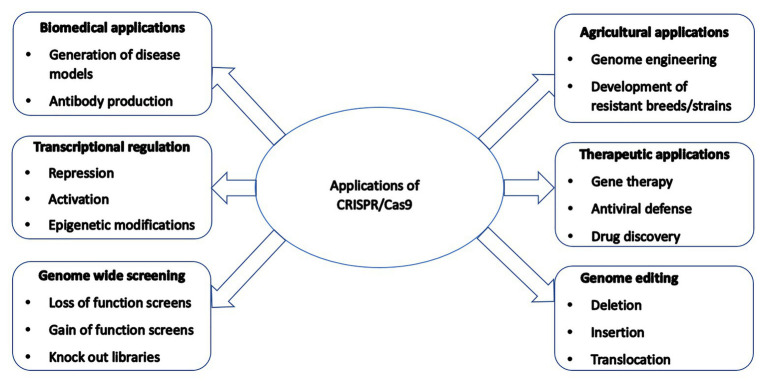
A summary of various applications of the CRISPR/Cas9 system in animals many of which are yet to be tested in poultry species.

CRISPR/Cas9-mediated genetically modified poultry-related species have many applications in agricultural and biomedical research. There is a steady upward trend in the number of published reports on the use of CRISPR/Cas9 gene editing technology in poultry species since its introduction a few years ago. Table 1 contains a selective list of the advances of CRISPR/Cas9-mediated gene edited poultry species and avian cells. This list was selected from recently published reports partly because of their significance on various aspects of CRISPR/Cas9-mediated genome editing in avian species, which is described in this review. Figure 2 shows a summary of various applications of the CRISPR/Cas9 system in animals many of which are yet to be tested in avian species.

**Table 1:** A selective list in advances of CRISPR/Cas9-mediated gene editing in poultry species and avian cells for different purposes.

**Genetic Modification in Avian Cells**	Target gene/Receptor	References
CRISPR mediated somatic cell genome engineering in the chicken	Paired Box 7 (PAX7)	Véron et al., 2015
Site-directed genome knockout in chicken cell line and embryos using CRISPR/Cas gene editing technology	C2EIP	Zuo et al., 2016
CRISPR/Cas9-mediated genome modification in chicken cell lines (B cell and DT40 cell lines)	DROSHA, DICER, MBD3, KIAA1279, CDKN1B, EZH2, HIRA, TYRP1, STMN2, RET, and DGCR	Abu-Bonsrah et al., 2016
Chicken cell line (DF-1) expressing edited PPAR-γ, OVA, ATP5E using CRISRP/Cas9 vectors	Peroxisome proliferator-activated receptor-γ (PPAR-γ), ATP synthase epsilon subunit (ATP5E), and ovalbumin (OVA)	Bai et al., 2016
Chicken DF-1 cells expressing myostatin gene knockout mediated by Cas9-D10A nickase without off-target effects	Myostatin	Lee et al., 2016
Targeted deletion mutation using poultry-specific CRISPR/Cas9 system in quail muscle cell line	Melanophilin (MLPH) locus	Ahn et al., 2017
Induced loss-of-function *via* a frameshift mutation in the CXCR4 gene in chicken PGCs	C-X-C chemokine receptor type 4 (CXCR4)	Lee et al., 2017c
CRISPR/Cas9-mediated chicken Stra8 gene knockout in male germ cell differentiation	Stimulated by retinoic acid 8 (Stra8) gene	Zhang et al., 2017
CRISPR/Cas9-mediated genome modulation of cis-regulatory interactions and gene expression in the chicken embryo	Msx1, Pax7, Sox9, c-Myb and Ets1	Williams et al., 2018
Chicken DF-1 cells expressing eGFP under control of the chicken GAPDH promoter	Glyceraldehyde-3-Phosphate Dehydrogenase (GAPDH) gene	Antonova et al., 2018
Genetic resistance to Avian Leukosis Viruses induced by CRISPR/Cas9 editing of specific receptor genes in chicken DF-1 cells	tva, tvc, and tvj receptor genes	Koslová et al., 2018
CRISPR/Cas9-Mediated TBK1 gene knockout chicken DF-1 cells	TANK binding kinase 1 (TBK1)	Cheng et al., 2019
HMEJ-mediated efficient site-specific gene integration in chicken DF-1 cells	Deleted in AZoospermia-Like (DAZL) gene	Xie et al., 2019
Direct delivery of adenoviral CRISPR/Cas9 vector into the blastoderm for generation of targeted gene knockout in quail	Melanophilin (MLPH) gene	Lee et al., 2019c
Sequential disruption of ALV host receptor genes in chicken DF-1 cells	tva, tvb, and chicken Na^+^/H^+^ exchange 1 (chNHE1) genes	Lee et al., 2019a
Functional study of the ANP32A genes mediated by the CRISPR/Cas9 system in chicken cell lines	Acidic (Leucine-Rich) Nuclear Phosphoprotein 32 Family, Member A (ANP32A)	Park et al., 2020
**Genetic Modification in Poultry Species**		
Chicken expressing CRISPR/Cas9-mediated OVA and OVM mutations	Ovalbumin (OVA) and ovomucoid (OVM)	Oishi et al., 2016
Chicken expressing CRISPR-targeted locus in PGCs	Immunoglobulin heavy chain locus of EGFP gene	Dimitrov et al., 2016
Chick embryo optimized for early loss-of-function using CRISPR/Cas9	Pax7 and Sox10	Gandhi et al., 2017
Chicken Embryo expressing CRISPR/Cas9	Somatic cells genes	Morin et al., 2017
Induced loss-of-function *via* a frameshift mutation in the CXCR4 gene in chicken PGCs	C-X-C chemokine receptor type 4 (CXCR4)	Lee et al., 2017c
Chickens overexpressing human IFN-β	Ovalbumin (OVA)	Oishi et al., 2018
Chicken primordial germ cells expressing gene insertion into Z chromosome for avian sexing model development	Z chromosome	Lee et al., 2019b
Efficient knock-in at the chicken ovalbumin locus using adenovirus as a CRISPR/Cas9 delivery system	Ovalbumin (OVA)	Qin et al., 2019
Precise CRISPR/Cas9 editing of the NHE1 gene renders chickens resistant to the J subgroup of avian leukosis virus	NHE1 gene	Koslová et al., 2020
Single amino acid deletion in myostatin propeptide of Japanese quail using CRISPR/Cas9	Myostatin (MSTN) gene	Lee et al., 2020
Acquiring resistance against a retroviral infection *via* CRISPR/Cas9 targeted genome editing in a commercial chicken line	Chicken Na^+^/H^+^ exchanger type 1 (chNHE1) receptor	Hellmich et al., 2020

### Agricultural Applications of CRISPR/Cas9 System in Poultry

Various agricultural traits can be achieved using CRISPR/Cas9-mediated gene editing approaches in poultry. Disease outbreaks in poultry pose a significant risk to the commercial poultry industry causing an increased cost of production for commercial poultry producers. There is a high demand for genetically modified chickens that are highly resistant to a specific disease-causing microorganism, and the available genome editing tools could help in this endeavor (Sid and Schusser, 2018). Avian influenza virus (AIV) is a poultry disease with high hypervirulence that causes sporadic pandemic events that lead to a high mortality rate (Suarez, 2000). Most vaccination strategies to control AIV are ineffective hence the need to breed resistance to AIV (Doran et al., 2017). There have been several recent attempts to suppress the transmission of AIV in genetically modified chickens. Lyall and his group generated transgenic chickens expressing a short-hairpin RNA (shRNA) that targets the viral genome. The shRNA is designed to inhibit and block influenza virus polymerase hence interfering with virus propagation, (Lyall et al., 2011).

Recent findings on the species-specific host co-factor polymerase activity of avian influenza viruses in chickens show that adding approximately 33 amino acid inserts in the chicken acidic nuclear phosphoprotein 32 family member A (chANP32A) protein enhances avian polymerase activity in avian cells. CRISPR/cas9 can also be used to substitute the chANP32A gene with huANP32A that has enhanced avian polymerase activity in avian cells. This could impair the enhanced polymerase activity of the avian influenza virus in chicken cells, thereby providing resistance to poultry species against influenza (Long et al., 2016). More recently, Park et al. (2020) conducted a study targeting chicken ANP32A using CRISPR/Cas9-mediated genome editing to examine the functional roles of ANP32A and other members of the ANP32 family using avian cell lines. The absence of the retinoic acid-induced gene I (RIG-I) in avian species has been shown to increase the susceptibility of chickens against AIV infection as compared to ducks where it is present hence making the ducks more resistant to influenza viruses (Barber et al., 2010). CRISPR/Cas9 can be used to introduce RIG-I-like disease-resistant genes in the genomes of poultry related species then breed these birds having higher resistance to AIV (Smith et al., 2015; Blyth et al., 2016). More recent studies conducted by Byun et al. (2017) have established the possibility to suppress AIV transmission in genetically modified birds that express the 3D8 single chain variable fragment (scFv).

Another poultry disease that causes economic losses in the poultry industry is the avian leukosis virus (ALV). ALV is a retrovirus that causes tumors in avian species by inserting a copy of their genome DNA into the host cell. Kučerová et al. (2013) identified W38 as the critical amino-acid residue in chicken Na^+^/H^+^ exchange 1 receptor (NHE1), whose deletion might confer the resistance to subgroup J avian leukosis virus. Lee et al. (2017a) were able to induce acquired resistance to ALV-J infection by using the CRISPR/Cas9-mediated homologous recombination in cultured chicken DF-1 cells. Lee et al. (2017c) modified critical residues of chicken NHE1 in cultured cells to induce resistance to viral infection and create mutations of the tryptophan residue at position 38 (Trp38) using single-stranded oligodeoxynucleotide (ssODN) recombination to confer resistance to ALV-J. In another research by Koslová et al. (2018), genetic resistance to ALV was successfully induced using the CRISPR/Cas9-mediated approach. Some frame-shifting mutations were introduced into tva, tvc, and tvj loci encoding receptors for the A, C, and J ALV subgroups, respectively. Therefore both Lee et al. (2017a) and Koslová et al. (2018) successfully produced KO or gene edits of NHE1 in the chicken DF-1 cell line. Lee et al. (2019a) used a CRISPR/Cas9-based disruption strategy of exon 2 within the tumor virus locus A gene (tva) of DF-1 fibroblasts to confer resistance to infection by ALV subgroup A. More recently, Koslová et al. (2020) prepared CRISPR/Cas9-mediated gene-edited chickens and found out that gene editing of the NHE1 gene renders chickens’ resistance to the J subgroup of avian leukosis virus. Therefore, Koslová et al. (2020) were able to produce an ALV-J-resistant chicken line as the first example of true site-specific gene editing. Hellmich et al. (2020) corroborated this strategy in commercial chicken lines by precise deletion of chicken NHE1 W38 using CRISPR/Cas9-system in combination with homology directed repair to induce ALV-J resistance. These examples show that CRISPR/Cas9 genome editing technology can be used widely to modify poultry species to produce a line of birds that exhibit desired resistance characteristics to viral infection. This might be the initial step in developing a virus-resistant line of birds in poultry. The use of such CRISPR-mediated genome edited poultry could substantially reduce a lot of economic losses as well as decreasing the cost of production in the poultry industry.

Increasing the performance of birds by enhancing muscle growth is another important agricultural application of CRISPR/Cas9-mediated gene editing in poultry species. MSTN suppresses skeletal muscle development and growth in animals (McPherron et al., 1997). A mutation in myostatin has resulted in increased muscle mass in mammals and fishes. In poultry, the increasing growth performance of birds can be enhanced by targeting MSTN to suppress its inhibitory effects on muscle growth. For example, a non-frameshift mutation in the MSTN of Japanese quail resulted in a significant increase in body weight and muscle mass (Lee et al., 2020). A disruption or removal of MSTN by genetic mutations using CRISPR/Cas9 inhibits its anti-myogenic function resulting in increased muscle mass in MSTN knockdown chickens (Bhattacharya et al., 2019). This is an important agricultural application in the poultry industry that could enhance bird performance and increase productivity, and help solve food shortage problems.

### Applications of CRISPR/Cas9 in Biomedical Research

Genome editing is a major development in biomedical research, with the current trend of innovative approaches providing directions for the treatment of various genetic and non-genetic diseases in the future. The availability of the CRISPR/Cas9-mediated gene and genome editing system has enabled the advent and use of more efficient strategies in gene targeting and the creation of gene edited avian species. This has guided recent and on-going advancements in biomedical research in the animal biotechnology field.

CRISPR/Cas9 technology has ushered in an innovative era in genome editing technology for the manipulation of invaluable avian models such as chickens. By applying CRISPR/Cas9 gene editing technology, researchers will be able to create an efficient bioreactor system for producing valuable proteins in poultry species. In chickens, the bioreactor system will enable efficient production and easy purification of egg white protein in large amounts (Lillico et al., 2005). The development of chickens as bioreactors for the production of target proteins has mostly utilized ovalbumin promoters (Park et al., 2015). The development of transgenic hens for protein production in eggs is highly necessary for the expression of therapeutic proteins which has resulted in significant advances in the generation of transgenic chicken models in this advancing era of genome editing. Oishi and colleagues have shown recently that the human interferon beta (hIFN-β) can be integrated into the chicken ovalbumin locus used in the production of hIFN-β in egg white (Oishi et al., 2018). Oishi et al. (2016) used CRISPR/Cas9 technology to demonstrate that disruptions of ovalbumin and ovomucoid genes had the potential to produce low allergenicity in eggs, which allowed a reduced immune response in egg white sensitive individuals. Therefore CRISPR/Cas9-mediated genome editing is expected to be key in the mitigation of allergic reactions caused by chicken eggs in some individuals by ensuring that chicken meat and eggs are allergen-free. This can be achieved by knocking out allergen-related genes such as ovalbumin and ovomucoid. This type of progress is important in the production of safe food products as well as the production of vaccines in the pharmaceutical industry.

The production of therapeutic antibodies against antigens is now possible through humanized chicken for therapeutic applications. The *loxP* site was inserted into the variable region of the immunoglobulin heavy chain using the CRISPR/Cas9-mediated approach (Dimitrov et al., 2016). Production of these genome-edited chickens will provide numerous opportunities for the discovery of therapeutic antibodies: a game-changer in biomedical research.

## Limitations of Using CRISPR/Cas9 System in Poultry Production

Despite the many advantages and breakthroughs that CRISPR/Cas9 system offers the poultry industry, several concerns touch on the ethical, legal, and social issues that affect the use of this powerful genome editing tool. One big concern of using the CRISPR/Cas9 technology is that this system generates off-target effects that can be very harmful. Off-target effects could play a critical role in the recognition and destruction of hypervariable viral nucleic acids or the plasmid DNA of beneficial bacteria that can potentially alter the microbiome profiles of a bird. With the newly developed ways of delivering the DNA-editing tool CRISPR-Cas9 into microorganisms, there is a possibility of altering the birds’ microbiome composition just like in other organisms (Hamilton et al., 2019; Ramachandran and Bikard, 2019). The cutting frequency determination (CFD) score of up to 0.28 has been found in some cases (Oishi et al., 2016; Koslová et al., 2020). The CFD score range from 0 to 1, with a higher off-target score, has much off-target potential that should be avoided. Off-target effects create unfavorable mutations at random sites that impact the precision of genome modification which raises concerns about safety and efficacy especially when the birds are raised for meat and egg production (Zhang et al., 2015; Chira et al., 2017).

There are high chances of having targeted alleles carrying additional modified and integrated targeted vectors through deletions and duplications because the DNA repair system has a scope that cannot integrate DNA fragments in the genetic makeup of an organism. This is based on the fact that the molecular mechanism that is used in the insertion of DNA fragments is highly mediated by the DNA repair mechanism that is turned on by the DSB created by the Cas9 enzyme (Li et al., 2015).

Decreasing the off-target effects may cause an upward trend in future applications of CRISPR/Cas9 gene-editing technology, especially in the generation of food animals such as poultry (Kleinstiver et al., 2016; Lee et al., 2017b). This goal could be achieved through studies that develop understanding of off-target mechanisms. The advent of transcriptome sequencing technology and the availability of high-throughput sequencing technology screening of gene edited animals can be enhanced to provide critical information about the potential off-targets associated with the use of CRISPR/Cas9 system in food animals (Roy et al., 2018).

Another major disadvantage of using the CRISPR/Cas9 system in poultry production is the low transfection efficiency (<2%) of avian cells in genome editing (Tyack et al., 2013; Lambeth et al., 2016) and the low germ-line transmission efficiency of less than 10% (Cooper et al., 2017; Hwang and Han, 2018). Just like other genome editing tools (TALENs and ZFNs), CRISPR/Cas9 system needs much more improvement to increase transfection efficiency and germ-line transmission. In the years before the advent of CRISPR technology, there were attempts to generate transgenic chickens but the germ-line transmission rate from one generation to another was very low. In Mozdziak et al. (2003) research group reported the first credible study of a genetically modified line of chickens that express a protein ubiquitously (Mozdziak et al., 2003). In Mozdziak et al. (2006) and his colleagues evaluated germline transmission rates of PGCs using fluorescence-activated cell sorting (Mozdziak et al., 2006). Many studies discussed earlier involving in ovo electroporation of chicken embryo proved to be very inefficient for germline transmission. There is a high possibility that the issue of low germline transmission efficiency in the production of genetically modified birds can be improved through PGC-mediated transgenesis and genome editing. First, PGCs are transfected then followed by subsequent injection into a host animal. The germline transmission rates obtained here are quite acceptable though they are variable from 0–90%. This could be an alternative strategy for improving germline transmission efficiency (Dimitrov et al., 2016).

Trends in the current meat market show that there are difficulties in the commercialization of transgenic poultry products generated by CRISPR/Cas9 technology in various countries around the world. This is mainly because of the high cost of developing this system and the major constraints of regulatory agents on genetically modified organisms (Manghwar et al., 2019).

## Current Strategies For Minimizing Off-Target Effects in CRISPR/Cas9-Mediated Genome Editing

### Improved Cas9 Variants

The most broadly utilized Cas9 is the *Streptococcus pyogenes* Cas9 (SpCas9), but it has been found to generate genome-wide off-target mutations. In the last 5 years, scientists have been working to develop Cas9 variants and other Cas9 orthologous that show minimized off-target effects and increased specificity to solve this issue. Among these, the available Cas9 variants include SaCas9, SpCas9-Nickase, dCas9, dCas9-FokI, xCas9, Cas9-NG, evoCas9, SpCas9-HFI, eSpCas9, Hypa-Cas9, Sniper-Cas9, HiFi Cas9, SpG, and PAM-less SpRY.

SaCas9 is a nuclease derived from *Streptococcus aureus*. It is widely used for ex vivo or *in vivo* gene therapy instead of SpCas9 due to its small size, which allows packaging in adeno-associated-virus (AAV) vectors. The saCas9 also recognizes a longer PAM sequence (5'-NNGRRT-3') as opposed to the shorter 5'-NGG-3' sequence recognized by SpCas9. Using SaCas9 for genome editing may therefore have very minimal off-target mutations (Kumar et al., 2018). Genome-wide unbiased identification of DSBs enabled by sequencing (GUIDE-seq) performed to detect off-targets show that the on-target activity was higher in the saCas9 than the wild type SpCas9 (Ono et al., 2019). SpCas9 nickase which is engineered through deactivation of the RuvC domain of SpCas9 through mutation has shown to have reduced off-target effects by more than 1,500 folds when compared with the wild type SpCas9 (Frock et al., 2015). dCas9-FokI which is deactivated or simply dead SpCas9 fused with the catalytic domain of FokI has shown decreased off-target sites and increased on-target activity by 140-fold when compared with the wild type SpCas9 (Wyvekens et al., 2015). XCas9, Cas9-NG, and evoCas9 is another set of engineered variants of spCas9 that have shown minimized off-target effects minimized and increased specificity in both animals and plants. The variant xCas9 recognizes a broad range of PAMs including GAT, GAA, and NG. Therefore, compared to SpCas9, xCas9 has a higher specificity and low off-target effects in animal cells (Liang et al., 2015; Hu et al., 2018). The GUIDE-seq has been used to assess the efficiency of Cas9-NG and evoCas9 at different loci. The on-target activity was significantly higher than off-target activity in both Cas9-NG (Nishimasu et al., 2018) and evoCas9 (Kleinstiver et al., 2015) than the wild type SpCas9. Other SpCas9 variants such as SpCas9-HFI (Kleinstiver et al., 2016), eSpCas9 (Slaymaker et al., 2016), Hypa-Cas9 (Chen et al., 2017), Sniper-Cas9 (Lee et al., 2018), HiFi Cas9 (Vakulskas et al., 2018), SpG and PAM-less SpRY (Walton et al., 2020) have been used more recently to minimize genome-wide off-target effects with exceptional accuracy.

### Improved Viral and Non-Viral CRISPR Delivery Methods

Viral vector delivery systems have been extensively used to deliver the components of gene-editing in gene therapy. In the CRISPR/Cas9 gene-editing system that uses viral based delivery methods, the Cas9 and gRNA are packaged into plasmid DNA, which is delivered *via* the viral vector to the target cell. This delivery increases the chances of off-target effects since the CRISPR/Cas9 components exist persistently in the target cell resulting in elevated Cas9 levels. Adeno viruses (AdV) have been used in viral vector delivery systems to minimize off-target effects since AdV show very minimal potential to integrate into the target cell genome (Gaj et al., 2017; Lino et al., 2018).

The non-viral delivery system involves directly delivering a ribonucleoprotein (RNP), which consists of the Cas9 protein in complex with a targeting gRNA to the target cells. The main advantage of this method is that RNPs may limit the potential for off-target effects since the Cas9-gRNA RNP is degraded over time (Vakulskas and Behlke, 2019). Minimized off-target mutations are possible when RNP complexes are delivered by liposome-mediated transfection as opposed to plasmid DNA transfection (Liang et al., 2015).

### Base Editing

NHEJ can introduce DSBs at unintended positions to the target gene hence generating insertions and deletions that are off targets. This causes off-target effects. Recently, a new genome-editing technique has been developed for base editing. This technique can change specific nucleotides in the genome without the introduction of double-stranded (ds) DNA breaks (Komor et al., 2016, 2018; Naeem et al., 2020). Base editing technique comprises of dCas9, catalytic base modification enzyme (deaminase), and sgRNA. The two categories of base editors developed recently are Cytosine base editors (CBE) and Thymine base editors (TBE) which can change C/G to T/A and A/T to G/C, analogously. The use of base editing has enabled new capabilities and applications in the genome editing world despite its recent introduction because it shows significant gene editing efficiency (Rees and Liu, 2018). An efficient base editing delivery system enhances the reduction of off-target mutations (Zhou et al., 2019).

### Prime Editing

Recently, Anzalone et al. (2019) reported that the development of a novel genome editing experimental approach that mediates all possible base-to-base conversions, “indels,” and combinations in mammalian cells without the need of a double-strand break or donor DNA (dDNA) templates. This new gene-editing method is called prime editing. Transition mutations by base editing are limited to installing four transition mutations efficiently, that is, C to T or G to A, A to G, and T to C. This strategy can therefore only make four of the 12 possible base pair changes. However, Prime editing can install all 12 possible transition changes (C/A, C/G, G/C, G/T, A/C, A/T, T/A, and T/G) in the genome. The prime editing system offers a new approach to minimizing off-target effects and increasing target specificity in genomes but requires more research on animal models to move it into therapeutic gene editing or for human consumption (Anzalone et al., 2019).

### Anti-CRISPR Proteins

The recent discovery of the protein inhibitors of CRISPR/Cas systems, called anti-CRISPR (Acr) proteins, has enabled the development of more efficient, controllable, and precise CRISPR/Cas tools in animal cells (Marino et al., 2020). More than 50 anti-CRISPR proteins have now been characterized up to date, each with its own means of blocking the cut-and-paste action of CRISPR systems (Dolgin, 2020). AcrIIA2 and AcrIIA4 proteins have been found to inhibit the CRISPR/Cas system and are hence desired to decrease off-target modifications without decreasing on-target activities in cells (Shin et al., 2017; Basgall et al., 2018).

## Future Perspectives

CRISPR/Cas9 technology has increased significantly the efficiency of the gene editing process when compared to the other modern existing processes of homolog recombination. CRISPR/Cas9-mediated gene editing is more advanced in small mammals such as mice and big mammals such as pigs than in avian species such as chickens, but very soon gene editing in poultry will enter into a highly competitive era of genome editing. In the future, the generation of poultry species expressing Cas9 will be beneficial to the study of biological processes. Studies of biological processes that enable us to understand the functions of the genes that may be involved in growth will be faster and easier in the future. This is already being done in pigs (Wang et al., 2017a) and can be utilized in poultry. In addition, the use of CRISPR/Cas9 to target PGCs offers a promising method of generating genetically engineered avian species with any desired gene characteristics (Abu-Bonsrah et al., 2016).

We predict that the future of the poultry meat industry will involve the production of birds that are highly efficient in feed utilization and lean meat which make them even more attractive for human consumption. Although the possibility of decreasing feed to gain ratio in poultry may be very minimal, this could change with the production of CRISPR-mediated transgenic chickens. There has been tremendous progress in the production of other meat animals such as pigs, with decreased fat deposition using the CRISPR/Cas9 system. For example, Zheng and his research group in China reconstructed the uncoupling protein 1 (UCP1) gene using CRISPR/Cas9 technology in the white adipose tissue of swine species, hence decreasing the accretion of fat (Zheng et al., 2017). In their study, Zheng and colleagues efficiently inserted a mouse adiponectin-UCP1 into the porcine endogenous UCP1 locus. The UCP1 knock-in pigs that were generated showed a decreased deposition of fat and increased carcass lean percentage. In poultry, the use of the CRISPR/Cas9 system has only recently taken off and is currently being used in targeting candidate avian genes in poultry species to produce birds that have higher lean meat and less fat which may lead to increased consumption by consumers (Park et al., 2019).

The production of foreign proteins in eggs can be utilized for industrial and therapeutic applications. Novel methods such as site-directed integration have been used by biotechnology companies such as AviGenics Incorporated (Athens, Georgia) and Crystal Bioscience Incorporated (Emeryville, California) to successfully create transgenic poultry for use in the production of biopharmaceutical proteins. Newer and innovative technologies such as CRISPR/Cas9 can further improve the efficiency of the production of these proteins. With the availability of CRISPR/Cas9 technology, cell and animal transgenesis providing a more efficient strategy through gene targeting and the creation of transgenic birds that will lead to advancements in biomedical research applications. Antibody-producing companies can purify overexpressed human antibodies from the eggs of poultry species such as chicken and quail to produce recombinant proteins and vaccines using CRISPR/Cas9-mediated approaches (Farzaneh et al., 2017). Furthermore, the production of antibodies using poultry eggs by utilizing the CRISPR/Cas9 system represents an economical and stress-free method of producing specific antibodies for therapeutic applications (Amro et al., 2018).

A great deal of time and resources are required before the CRISPR-Cas9 system becomes 100% safe and effective in the generation of food animals. If the remaining safety and efficiency concerns are fully addressed, then the CRISPR/Cas9 system could be effectively used to improve food quality and production. Diversity among the poultry species should be strongly encouraged and pursued using gene editing technologies. However, because the resulting birds will be genetically engineered and modified, the Food and Drug Administration (FDA) will have to review and approve the use of such poultry birds after guaranteeing that the meat and eggs produced are safe for human consumption. It is expected that in the near future, CRISPR/Cas9-mediated genome editing research will extend to other categories of poultry species such as turkeys, geese, ducks, and guinea fowl across the world since major progress has been made in chicken and quail.

Several recent trends might fast-track the generation of transgenic birds in the near future. First, *in vitro* genetically manipulated PGCs could be re-introduced not only into the embryonic blood but also into the testes of sterilized adult recipients. After such transplantation, donor PGCs colonize the spermatogenic epithelium and mature into fertile sperm. This method was recently described by Trefil et al. (2017). Compared with existing approaches, this procedure will become the method of choice in the future because it is more efficient, faster, requires fewer animals, and could broaden PGC technology in other poultry species. Secondly, genetic sterility might be a very useful tool for CRISPR/Cas9-assisted gene editing. Genetically sterile chickens can be used as surrogate hosts for germ line transfer (Woodcock et al., 2019) or, in the future, for efficient transgenesis. Finally, the use of adenoviral vectors for CRISPR/Cas9 delivery could bring the technique of virus subgerminal injection back into routine use (Lee et al., 2019c). The implementation of this method could accelerate avian knockout studies and lead to the advancement of future agricultural applications.

## Concluding Remarks

The development and improvement of CRISPR technology over the years has enabled access to generate transgenic lines of birds for meat or egg production, mainly for food. The impact of CRISPR technology could potentially lead to the efficient improvement and sustainability of poultry products, which will help address challenges associated with universal food security. Birds raised for meat and egg production using the CRISPR technology could have an immense impact on the advancement of poultry related traits such as feed conversion, digestibility, increased egg production, growth, and overall improved performance of birds. Innovations resulting from CRISPR technology could also lead to developments in fields such as disease resistance, immune function, and vaccine delivery. This will in turn enhance poultry health, increase the safety of vaccines produced using chicken eggs, and increase food safety and production.

The future applications of CRISPR technology in poultry have promising and tremendous potentials in biomedical research that could benefit humankind due to vast opportunities for disease treatment and prevention. Most of these applications have been focused on chickens that show great potential for biomedical research. Finally, yet importantly, the latest progressions in CRISPR/Cas9 gene editing technologies might assist in scaling down or abolishing barriers such as the difficulties of gaining regulatory approval and the public perception and acceptability of CRISPR technology in the production of food animals.

## Author Contributions

CK wrote the first draft of the manuscript. CK and SN revised and approved the manuscript. All authors contributed to the article and approved the submitted version.

### Conflict of Interest

The authors declare that the research was conducted in the absence of any commercial or financial relationships that could be construed as a potential conflict of interest.
